# A spiking-neuron model of memory encoding and replay in hippocampus

**DOI:** 10.1186/1471-2202-15-S1-P166

**Published:** 2014-07-21

**Authors:** Oliver Trujillo, Chris Eliasmith

**Affiliations:** 1 Centre for Theoretical Neuroscience, University of Waterloo, Waterloo, Ontario, Canada N2L 3G1

## 

Hippocampal cells replay sequences of neural activity that have been experienced in the past, implicating the hippocampus in episodic memory. We present a spiking-neuron attractor network model that can encode arbitrary experiences in a combination of learned connection weights and neural activity. The model can then recall part or all of the encoded experience based on contextual cues, simulating hippocampal replay.

The simulation is run using the Neural Engineering Framework (NEF) [1] and the Nengo neural modelling tool [2]. It consists of 77740 simulated spiking LIF neurons, divided into populations representing hippocampal areas CA3 and CA1, and part of EC. The NEF provides a method for dynamically representing vector-values and computing transformations on them with neural populations.

Our model takes as input a sequence of high-dimensional vectors representing sensory information entering the hippocampus from EC. These vectors can represent position information (place cells) or any other form of processed sensory data. Strong recurrently connected populations in area CA3 cycle through a series of temporal indices, and a Hebbian learning rule allows for these indices to be uniquely generated for a given environment. Using the NEF to compute a binding operator [3], the network associatively binds the sensory input vectors with an index. These bindings are stored in the neural activity of the recurrent CA1-EC loop. The network can switch between *encoding* and *recall* modes, and when in recall mode can look up the corresponding index bound to the experience being recalled and, using the same indexing system in CA3, can complete the pattern of neural activity corresponding to its previously remembered experience.

**Figure 1:**
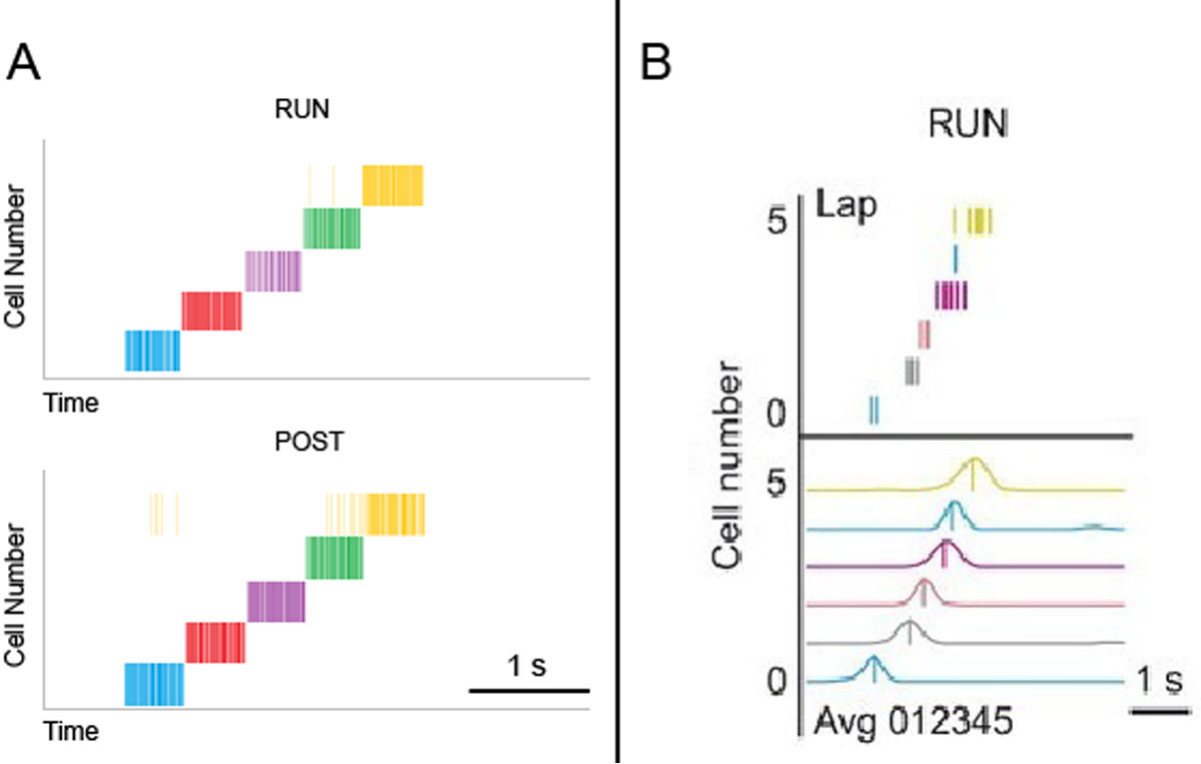
Neural spike data collected from the model (A) and rats (B) showing similar neural spike data during the experiment (RUN) and during recall (POST).

The model was run in encoding mode on a sequence of 5 different random HRR vectors in 64 dimensions over a period of 2.5 seconds. It was then given the first in the sequence of vectors as input. After 1 second of re-orientation, the model was switched into recall mode. Figure 1A shows spike trains from neurons in the model at a point in the original run and at the corresponding point in the recalled sequence. For comparison, figure 1B shows hippocampal recordings from rodents in [4].

To the best of our knowledge, this is the first spiking neural model to take into account both timing and sensory tuning of hippocampal cells, while exhibiting the ability to recall previously experienced sequences. In addition, it operates on arbitrary sensory vectors as input, thus not constraining the model to spatial or non-spatial information and allowing it to be extended to perform spatial navigation tasks.
